# The cAMP-producing agonist beraprost inhibits human vascular smooth muscle cell migration via exchange protein directly activated by cAMP

**DOI:** 10.1093/cvr/cvv176

**Published:** 2015-06-19

**Authors:** Jenny S. McKean, Fiona Murray, George Gibson, Derryck A. Shewan, Steven J. Tucker, Graeme F. Nixon

**Affiliations:** 1 School of Medical Sciences, University of Aberdeen, Aberdeen AB25 2ZD, UK; 2 Department of Cardiothoracic Surgery, Aberdeen Royal Infirmary, Aberdeen, UK

**Keywords:** Vascular smooth muscle cell, Migration, Prostacyclin, cAMP, Epac

## Abstract

**Aims:**

During restenosis, vascular smooth muscle cells (VSMCs) migrate from the vascular media to the developing neointima. Preventing VSMC migration is therefore a therapeutic target for restenosis. Drugs, such as prostacyclin analogues, that increase the intracellular concentration of cyclic adenosine monophosphate (cAMP) can inhibit VSMC migration, but the mechanisms via which this occurs are unknown. Two main downstream mediators of cAMP are protein kinase A (PKA) and exchange protein directly activated by cAMP (Epac). This study has examined the effects of the prostacyclin analogue beraprost on VSMC migration and investigated the intracellular pathways involved.

**Methods and results:**

In a chemotaxis chamber, human saphenous vein VSMC migrated towards a platelet-derived growth-factor-BB (PDGF) chemogradient. Incubation with therapeutically relevant concentrations of cAMP-producing agonist beraprost significantly decreased PDGF-induced migration. Direct activation of either PKA or Epac inhibited migration whereas inhibition of PKA did not prevent the anti-migratory effect of beraprost. Direct activation of Epac also prevented hyperplasia in *ex vivo* serum-treated human veins. Using fluorescence resonance energy transfer, we demonstrated that beraprost activated Epac but not PKA. The mechanisms of this Epac-mediated effect involved activation of Rap1 with subsequent inhibition of RhoA. Cytoskeletal rearrangement at the leading edge of the cell was consequently inhibited. Interestingly, Epac1 was localized to the leading edge of migrating VSMC.

**Conclusions:**

These results indicate that therapeutically relevant concentrations of beraprost can inhibit VSMC migration via a previously unknown mechanism involving the cAMP mediator Epac. This may provide a novel target that could blunt neointimal formation.

## 1. Introduction

The most common clinical interventions in coronary artery disease are balloon angioplasty followed by stent emplacement or coronary artery bypass graft. Both these treatments are associated with neointimal hyperplasia resulting in restenosis and vessel reocclusion.^1,2^ While advancements have been made in stent design, alternative drug strategies are required to significantly benefit patient outcomes.^3^ One of the essential cellular processes involved in neointimal hyperplasia is an increase in migration of VSMC from the media to the intima.^4^ This cellular process has yet to be successfully targeted in restenosis. At a cellular level, VSMC migration is initiated by growth factors such as platelet-derived growth-factor-BB (PDGF), cytokines (for example tumour necrosis factor-α), and extracellular matrix components.^4^ These mediators stimulate migration in a spatially and temporally organized process. Initially, a remodelling of the actin cytoskeleton occurs throughout the cell resulting in protrusion of a leading edge.^5^ This leading edge extends towards a chemotactic stimulus (for example along a PDGF concentration gradient), assisted by the actions of matrix metalloproteinases which degrade the extracellular matrix.^6^ Movement of the cell occurs via the actomyosin motors which generate force.^5^ The complex guidance cues and intracellular pathways which regulate this migration process in VSMC are still unclear. It is known that monomeric G-proteins have an integral and interdependent role in migration.^4,7^ In particular, the role of RhoA has been characterized in many cell types including VSMC.^7–9^ RhoA regulates actin polymerization via actin-binding proteins involved in focal contact formation with integrins as well as regulating the activity of myosin II.^10^ The actions of RhoA are mediated by Rho effectors such as p160ROCK (Rho-kinase) which can phosphorylate multiple target proteins.^7^

In addition to mechanisms which activate VSMC migration, there are endogenous mechanisms which maintain quiescence, thereby preventing migration. At the level of intracellular signalling, it is clear that increases in cyclic adenosine monophosphate (cAMP) can inhibit growth-factor-induced VSMC migration.^11^ Although currently untested, this approach could potentially indicate that cAMP-activating drugs may be useful in drug-eluting stents to decrease neointimal hyperplasia. Indeed, prostacyclin and its analogues have been recognized for some time to inhibit neointimal hyperplasia in animal models via a variety of mechanisms, including elevation of intracellular cAMP concentrations.^12–15^ The mechanisms involved in cAMP-mediated inhibition of VSMC migration are not clear. Until recently the downstream effects of increased cAMP were assumed to be predominantly via the well-described mediator, protein kinase A (PKA). However, cloning of the cAMP-binding protein, exchange protein directly activated by cAMP (Epac), has offered additional cAMP-dependent mechanisms with different downstream consequences.^16,17^ Epac has 2 known isoforms, Epac1 and Epac2, with little functional difference ascribed to their effects. Epac is one of a family of guanine nucleotide exchange factors for the monomeric G-protein Rap1.^17^ Rap1 has several different functions dependent upon cell type and is frequently associated with regulation of the cell cytoskeleton.^18^ The Epac-Rap1 pathway is intimately involved in regulating cell migration and cell-cell interactions in a cell type dependent manner.^19,20^ Both Epac/Rap1 and PKA may be involved in relaxation of smooth muscle and could inhibit VSMC proliferation, although their role in VSMC migration is still unknown.^21–23^

In the current study, we have examined VSMC migration and the effects of increasing cAMP using the prostacyclin analogue beraprost. We now demonstrate that at therapeutically relevant concentrations of beraprost, VSMC migration is inhibited via a divergent mechanism dependent on cAMP and Epac, but does not involve PKA. These findings indicate that cAMP-producing agonists such as beraprost may be useful in decreasing restenosis by preventing VSMC migration. The study also indicates Epac/Rap1 as a potential therapeutic target.

## 2. Methods

An expanded Methods section is available in Supplementary material.

### 2.1 Primary cell culture of human vascular smooth muscle cell

Saphenous veins were obtained from patients undergoing coronary artery bypass graft surgery after obtaining written consent under procedures approved by the local ethical committee (reference 06/S0802/26). These procedures conform to the principles outlined in the Declaration of Helsinki. VSMC were cultured from vascular explants and maintained as previously described. All cells used were between passages 3–8. For *ex vivo* organ culture, veins were incubated in 30% bovine calf serum for 14 days and medium changed every 48 h.

### 2.2 Chemotaxis assays

Cells were kept in serum-free medium for 24 h and seeded into a 48 multi-well chemotaxis chamber as per the manufacturer's instructions. Migrated cells bound to the underside of the membrane were mounted onto a microscope slide and the cells counted using ImageJ. All samples were completed in duplicate.

### 2.3 Bromodeoxyuridine cell proliferation assay

Cells were plated into 96-well plates in 0.1% FCS DMEM for 24 h. Treatment medium was added along with the bromodeoxyuridine (BrdU) label for a further 24 h. The BrdU assay was carried out as per manufacturer's instructions and repeated in triplicate.

### 2.4 SDS–PAGE electrophoresis/immunoblotting

Protein extracts were prepared and subjected to SDS–PAGE followed by immunoblotting as previously described.^24^

### 2.5 Immunofluorescence staining

Cells were serum starved for 24 h and, following treatment, were fixed in 3% paraformaldehyde as previously described.^24^ Cells were imaged using a Zeiss 710 scanning confocal microscope with a 40× objective. During confocal imaging comparing different cell treatments, settings on the microscope were not changed (including contrast, aperture, and laser strength). Experiments were repeated in triplicate.

### 2.6 siRNA transfection

Knockdown of specific proteins was carried out as previously described for human VSMC.^24^ Cells were transfected with either 37.5 nmol/L scrambled siRNA (Dharmacon) or 18.75 nmol/L of each PKAα and -β catalytic subunit siRNA (validated siRNA from Qiagen) or 18.75 nmol/L Rap1A and Rap1B SMARTpool siRNA (validated siRNA from Dharmacon) using Lipofectamine RNAiMAX (Invitrogen) according to the manufacturer's instructions. For sequence data, see Supplementary material online, *Table S1*. The cells were serum starved the following day for 24 h and then the protein was extracted for immunoblotting or the cells were used in the chemotaxis chamber.

### 2.7 Fluorescence resonance energy transfer

A human aortic smooth muscle cell nucleofector kit cc-2571 from Lonza was used to introduce the cDNA into the VSMC. For experiments investigating the Epac protein, 8 µg CFP-Epac-YFP cDNA was used (kindly donated by Dr. Kees Jalink, The Netherlands Cancer Institute, Amsterdam, The Netherlands). The PKA cDNA consists of a regulatory subunit construct and a catalytic subunit construct (kind gift from Prof. Manuela Zaccolo, University of Glasgow, Glasgow, UK). The ratio of the two PKA cDNA was optimised and 2 µg of the donor and 3 µg of the acceptor were added. Briefly cells were passaged and transferred to an electroporation cuvette with the cDNA and nucleofector solution and transfected according to manufacturer's instructions. The cells were plated in single glass-bottom dishes and following stimulation time-lapse videos of transfected VSMC were captured using a Leica AF6000LX imaging system which captures simultaneously in cyan fluorescent protein (CFP), yellow fluorescent protein (YFP), and FRET configured channels. Correction factors were entered into the software to correct for channel bleed-through and background fluorescence. The correction factors have previously been generated using control experiments described.^25^ Images of 378 ms duration were captured every minute with a Roper Coolsnap 1392 × 1040 pixel camera with 4 × 4 binning. The Leica 11504119 Fret EX filter separated the fluorescent channels: a CFP donor channel (excitation at wavelength 427 ± 5 nm and emission at 482 ± 15 nm), an FRET channel (excitation at wavelength 427 ± 5 nm and emission at 542 ± 13 nm), and a YFP channel (excitation at wavelength 500 ± 6 nm and emission at 542 ± 13 nm). These images were used to create a pseudocolour sensitized FRET image for each time point using specific algorithms.^25^ Regions of interest were traced using the Leica Advanced Fluorescence software and FRET values recorded. The FRET data are displayed as percentage change from the initial control readings before treatments were added at 5 min, with values >0% reflecting protein activation and values <0% reflecting protein inactivation. All experiments were performed in duplicate.

### 2.8 Rap1 and Rho pull-down assay

Pull-down assays in human VSMC were performed as previously described.^26^ Cells were plated in 75 cm^2^ flasks and grown to 80–90% confluent. The cells were serum starved for 24 h. After this time period, treatment medium was added to the flasks for 5–10 min for the Rap1 experiments and 30 min for the Rho experiments. After incubation the Rap1 or Rho pull-down assay was carried out as per manufacturer's instruction (Thermo Scientific). Briefly, the cells were scraped and centrifuged at 16 000 *g* for 15 min at 4°C. Two controls were used including the GTPγs (positive control) and GDP (negative control) throughout the assay. The lysates were placed into spin cups with a GST–fusion protein of the Rap1-binding domain from human RalGDS to pull down the active Rap1 protein or the Rhotekin-binding domain along with glutathione agarose resin to pull down the active Rho protein. Immunoblotting was then carried out with either an anti-Rap1 or anti-Rho antibodies for the detection of the GTP-bound active form of the protein.

### 2.9 Statistics

All experiments were performed with either three or more individual patient vein explants. Data are expressed as the mean ± SEM. For experiments that were performed in duplicate or triplicate, replicates were averaged and average values used to calculate the mean.

A paired *t*-test was performed for the Rap1 RT-PCR (supplementary material online, *Figure S2**B*) and *Figure 6D*, where the same cells at the same passage were used with different treatments. A two-way ANOVA was performed with Dunnett's post hoc test for the FRET data to compare each timepoint with different treatments. All other experiments were analysed with a one-way ANOVA with Dunnett's post hoc test. A *P* value of <0.05 was taken as statistically significant.

## 3. Results

### 3.1 Beraprost inhibits growth-factor-induced migration, but not proliferation, of human VSMC

**Figure 1:**
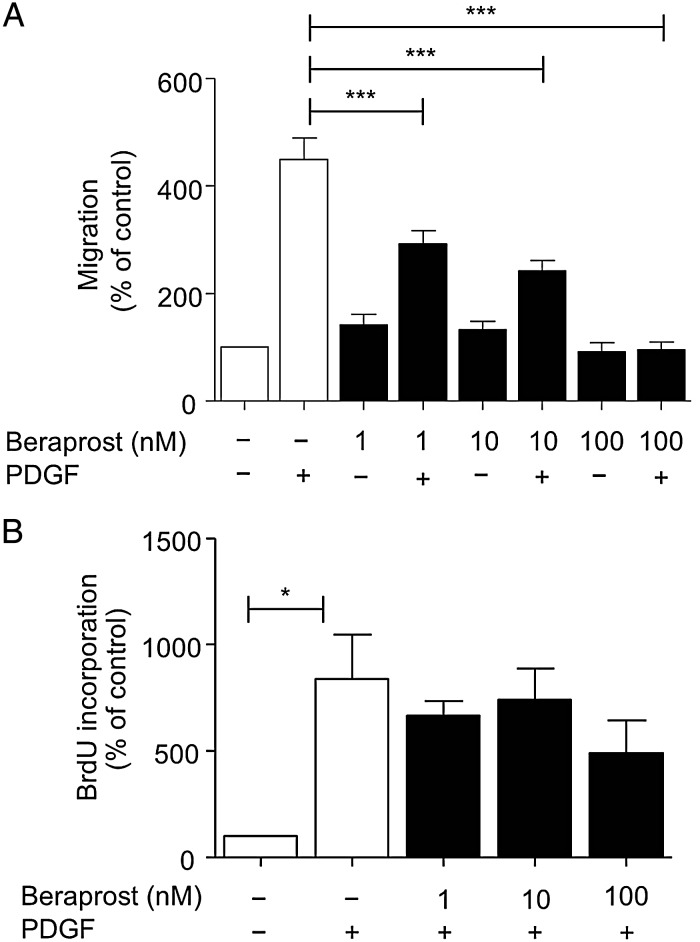
Beraprost inhibits migration in human VSMC but does not alter proliferation*.* (*A*) VSMC were seeded into the top well of a chemotaxis chamber and PDGF (10 ng/mL) was added with or without increasing concentrations of beraprost (1–100 nmol/L) to the bottom well. PDGF-induced migration was inhibited at all concentrations of beraprost examined, *n* = 6. (*B*) VSMC were treated with PDGF with or without beraprost for 24 h. Increasing concentrations of beraprost had no effect on PDGF-induced BrdU incorporation, *n* = 3. Data are shown as mean percentage ± SEM. ***P < 0.001, *P < 0.05.

To determine whether beraprost can inhibit human VSMC migration, primary cultured VSMC were seeded onto membranes in a modified Boyden chemotaxis chamber and incubated with beraprost. After 4 h in the chamber, cells had significantly migrated towards a chemogradient established by 10 ng/mL PDGF. Beraprost (1, 10 and 100 nmol/L) significantly inhibited VSMC migration (*Figure 1A*). Similar results were obtained with another prostacyclin analogue iloprost (not shown). To ensure that changes in migration were not due to the actions of beraprost on the mitogenic effects of PDGF, BrdU incorporation was assessed (*Figure 1B*). At 4 h, BrdU incorporation was not significantly increased by PDGF (not shown). Furthermore, at 24 h, although VSMC incubated with PDGF had significantly increased BrdU incorporation, the addition of beraprost did not significantly alter this effect.

### 3.2 PKA and Epac activation inhibit migration

**Figure 2:**
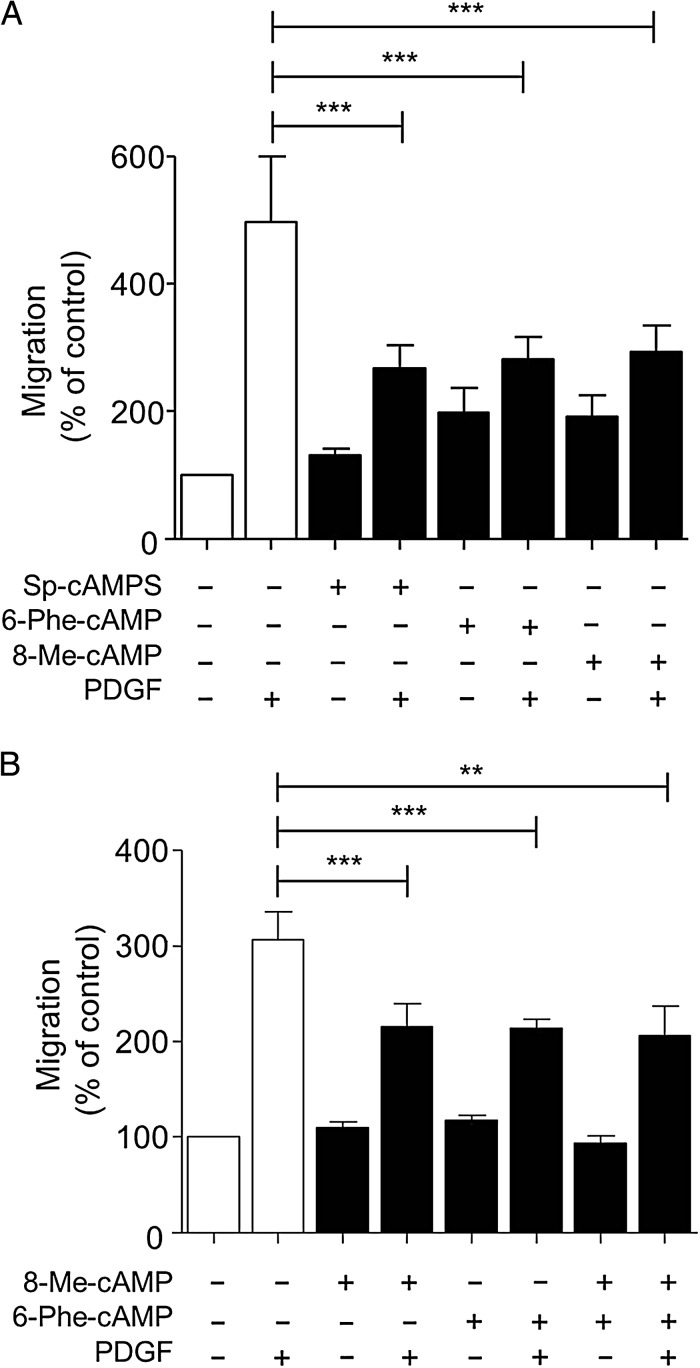
cAMP signalling can inhibit human VSMC migration via multiple mediators. VSMC were added to the chemotaxis chamber in the presence of PDGF (10 ng/mL). (*A*) PDGF-induced migration was inhibited in the presence of Sp-cAMPS (20 µmol/L), 6-Phe-cAMP (5 µmol/L), or 8-Me-cAMP (2 µmol/L), *n* = 5. (*B*) The simultaneous addition of 6-Phe-cAMP (5 µmol/L) and 8-Me-cAMP (2 µmol/L) in the presence of PDGF had no further effect on VSMC migration in comparison with 6-Phe-cAMP or 8-Me-cAMP alone, *n* = 5, **P < 0.01, ***P < 0.001. Data are expressed as mean ± SEM Percentage of migration in comparison to the control.

Since beraprost inhibits PDGF-induced VSMC migration, we investigated the downstream mediators involved. VSMC were incubated with cAMP analogues that preferentially activate either PKA or Epac and PDGF-induced migration was determined. VSMC were incubated with either 8-bromoadenosine 3′,5′-cyclic monophosphorothioate Sp-isomer (Sp-cAMPS) (Epac and PKA agonist), *N*^6^-phenyladenosine-3′,5′-cyclic monophosphate (6-Phe-cAMP) (selective PKA agonist) or 8-(4-chlorophenylthio)-2′-*O*-methyladenosine 3′,5′-cyclic monophosphate (8-Me-cAMP) (selective Epac agonist). All of these cAMP analogues inhibited PDGF-induced VSMC migration (*Figure 2A*). The effects of 6-Phe-cAMP and 8-Me-cAMP were not additive (*Figure 2B*) despite the concentrations used being submaximal (supplementary material online, *Figure S1*). Differences in the total migration achieved with PDGF can be observed in *Figure 2A* compared with *Figure 2B*. This is due to variation within the patient samples obtained.

### 3.3 At therapeutic concentrations, beraprost activates Epac but not PKA in VSMC to inhibit migration

**Figure 3:**
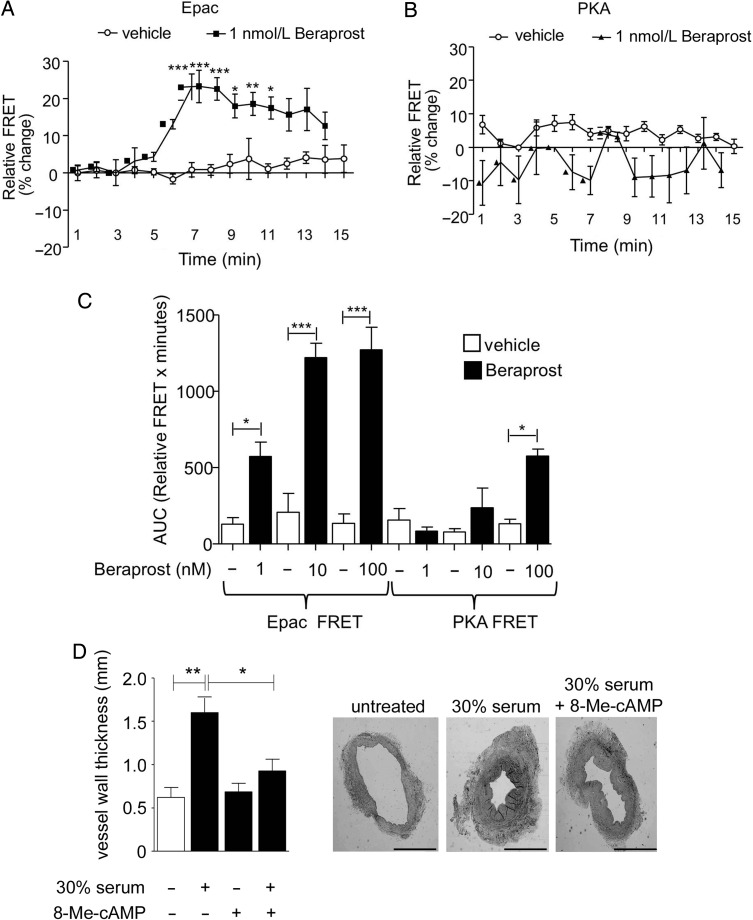
Beraprost at therapeutic concentrations activates Epac but not PKA. (*A*) The addition of 1 nmol/L beraprost (at timepoint 5 min) to VSMC transfected with the Epac sensor resulted in a time-dependent significant decrease in FRET activity (denoting an increase in Epac activation) compared with control (no beraprost added), *n* = 5. Data are expressed as percentage change ± SEM in FRET activity. (*B*) 1 nmol/L beraprost incubation in VSMC transfected with the PKA sensor produced no significant change in FRET activity compared with vehicle-treated cells throughout the timecourse examined (up to 60 min, 15 min shown), *n* = 6. Data are expressed as percentage change ± SEM in FRET activity, drug incubation starts at 5 min timepoint. (*C*) Bar graph showing area under the curve (AUC) for FRET activity during 60 min of beraprost incubation. Beraprost-treated cells (1, 10, and 100 nmol/L) are compared with their respective vehicle-treated controls, (*n* = 4–7. All three concentrations of beraprost significantly increased Epac activation whereas only the highest concentration examined (100 nmol/L) significantly increased PKA activation. (*D*) Vessel wall thickness of H&E stained sections from vessels cultured *ex vivo* for 14 days was measured using ImageJ and compared with sections from vessel segments fixed immediately for microscopy. Incubation with 50 µmol/L 8-Me-cAMP significantly decreased the serum-induced hyperplasia (*n* = 3 veins from different subjects). *P < 0.05, **P < 0.01, ***P < 0.001.

Pharmacokinetic studies of beraprost in humans have determined that the peak plasma concentration of beraprost which can be achieved is up to 345 ng/mL (∼1 nmol/L).^27^ This peak plasma concentration does not increase with multiple doses. To determine whether beraprost activates either Epac or PKA at this therapeutically relevant concentration, we transfected VSMC with fluorescence resonance energy transfer (FRET) sensors for Epac or PKA. Single cells were stimulated with either 1, 10, or 100 nmol/L beraprost and FRET activity for each probe was measured and expressed as relative FRET activity of the whole cell. 1 nmol/L beraprost induced a significant decrease in FRET activity (increase in sensor activation) of the Epac probe 1 min after incubation compared with vehicle-treated cells (drug added after 5 min of baseline measurement). This reached a peak between 2 and 5 min after beraprost addition and decreased thereafter (*Figure 3A*). However, 1 nmol/L beraprost did not change the activity of the PKA FRET probe in VSMC over 15 min, indicating that PKA is not activated at this concentration (*Figure 3B*). Additional experiments carried out over longer periods of up to 60 min duration also did not reveal any significant change in PKA activity as assessed by both the % change in FRET activity (not shown) or area under the curve (*Figure 3C*). Similarly, incubation with 10 nmol/L beraprost did not result in a change in PKA FRET probe activity compared with untreated cells over a 60 min timecourse (*Figure 3C*). Epac activation, however, was significantly increased over a 60 min timecourse by incubation with 10 nmol/L beraprost (*Figure 3C*). Notably, higher concentrations of beraprost (100 nmol/L) produced a significant increase in both Epac and PKA FRET activity in cells transfected with the respective probes (*Figure 3C*). To ensure that the observed differences between activity of the Epac and PKA FRET probes to beraprost were not due to different sensitivities of the different sensors, VSMC were incubated with increasing concentrations of forskolin, an adenylate cyclase activator and FRET activity was measured. Changes in the magnitude and timecourse of FRET activity were not significantly different comparing Epac and PKA probes in response to 1 and 10 µmol/L forskolin (supplementary material online data, *Figure S2*). These data suggest that Epac, but not PKA, is activated by a therapeutically relevant concentration of beraprost to inhibit VSMC migration.

In addition, we examined an *ex vivo* model of hyperplasia to determine whether Epac could prevent hyperplasia in blood vessels. Four sequential human saphenous vein segments (from the same vein) were either; (i) fixed immediately (control), (ii) cultured for 14 days in 30% FBS-containing medium, (iii) cultured for 14 days in serum-free medium with 50 µmol/L Epac analogue 8-Me-cAMP, or (iv) cultured for 14 days in 30% FBS-containing medium and 50 µmol/L Epac analogue 8-Me-cAMP. Subsequent analysis of saphenous vein paraffin-embedded sections subjected to H&E staining revealed that the serum-induced increase in vessel wall thickness was significantly reversed by incubation with the Epac analogue (*Figure 3D*).

### 3.4 Therapeutic concentrations of beraprost inhibit VSMC migration via an Epac-Rap-1 pathway

**Figure 4:**
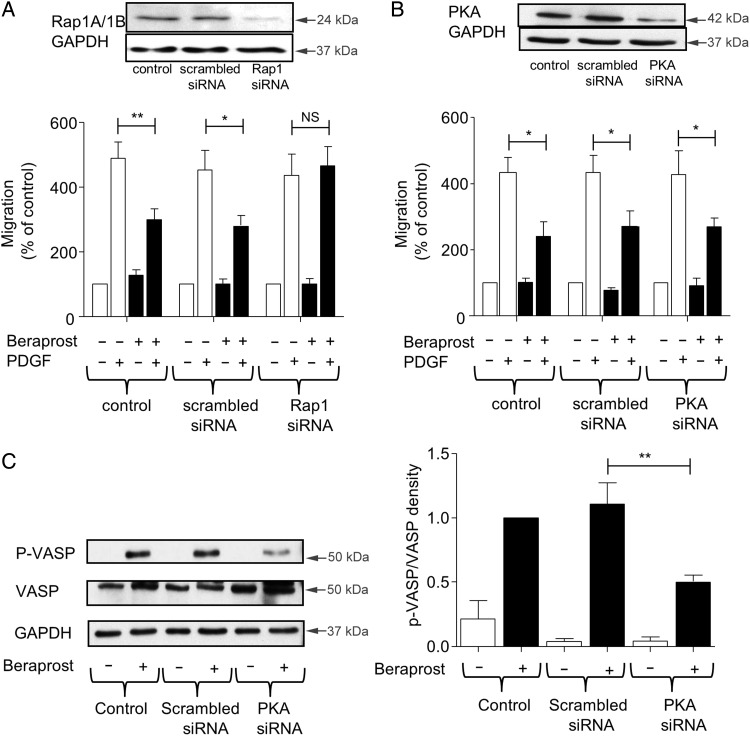
Inhibition of human VSMC migration by therapeutic concentrations of beraprost requires Epac/Rap1 activation but not PKA. (*A*) Transfection of Rap1 siRNA significantly decreased Rap1 protein in VSMC by 81% in comparison to scrambled siRNA. In the chemotaxis chamber, 1 nmol/L beraprost had no effect on PDGF-induced migration in Rap1 siRNA-treated VSMC. Data are expressed as mean percentage of migration in comparison to the control ± SEM, *n* = 4. (*B*) PKA siRNA to A and B catalytic subunits of PKA decreased PKA protein expression in VSMC by 74% in comparison to the scrambled siRNA. PKA siRNA-treated VSMC were added to the chemotaxis chamber. 1 nmol/L beraprost inhibited PDGF-induced migration despite decreased PKA expression. Data are expressed as mean percentage of migration in comparison to the control ± SEM, *n* = 4. (*C*) The effect of PKA siRNA on phosphorylation of the PKA downstream effector, VASP, was examined. VASP phosphorylation induced by 100 nmol/L beraprost (a concentration which activates both PKA and Epac) after 1 h incubation was significantly decreased in the PKA siRNA-treated VSMC compared with cells transfected with scrambled siRNA and control (untransfected) cells. Data are displayed as p-VASP/total VASP, mean ± SEM, *n* = 4. *P < 0.05, **P < 0.01, NS = non-significant.

An siRNA-mediated decrease in the expression of Epac1 and Epac2 was not possible in human saphenous vein VSMC (see Supplementary material online, Methods). Alternatively we targeted Rap1 expression. VSMC were incubated with siRNA-specific for Rap1A and Rap1B. Rap1 (both Rap1A and Rap1B) protein expression was significantly decreased by 81 ± 17% compared with cells incubated with scrambled siRNA (*Figure 4A*). This decrease in Rap1 protein expression had no significant effect on PDGF-induced VSMC migration as assessed using a modified Boyden chamber (*Figure 4A*). However, inhibition of migration by 1 nmol/L beraprost was significantly reversed in these cells compared with cells incubated with scrambled siRNA or control (untransfected) cells indicating the involvement of the Rap1 pathway in the anti-migratory effects of beraprost. To determine the potential involvement of PKA, VSMC were transfected with siRNA which resulted in 74 ± 11% decrease in PKA catalytic subunit protein expression (*Figure 4B*). This knockdown in PKA protein expression did not produce any change in either PDGF-induced migration or the inhibitory effect of 1 nmol/L beraprost (*Figure 4B*). To ensure that the level of decrease in PKA protein expression was sufficient to induce a functional change, we examined phosphorylation of vasodilator-stimulated phosphoprotein (VASP), a known downstream target protein of PKA. In cells transfected with scrambled siRNA, 100 nmol/L beraprost incubation (which activates PKA, *Figure 3E*) resulted in an increase in VASP phosphorylation within 60 min (*Figure 4C*). This increase was significantly inhibited in cells transfected with PKA siRNA.

**Figure 5:**
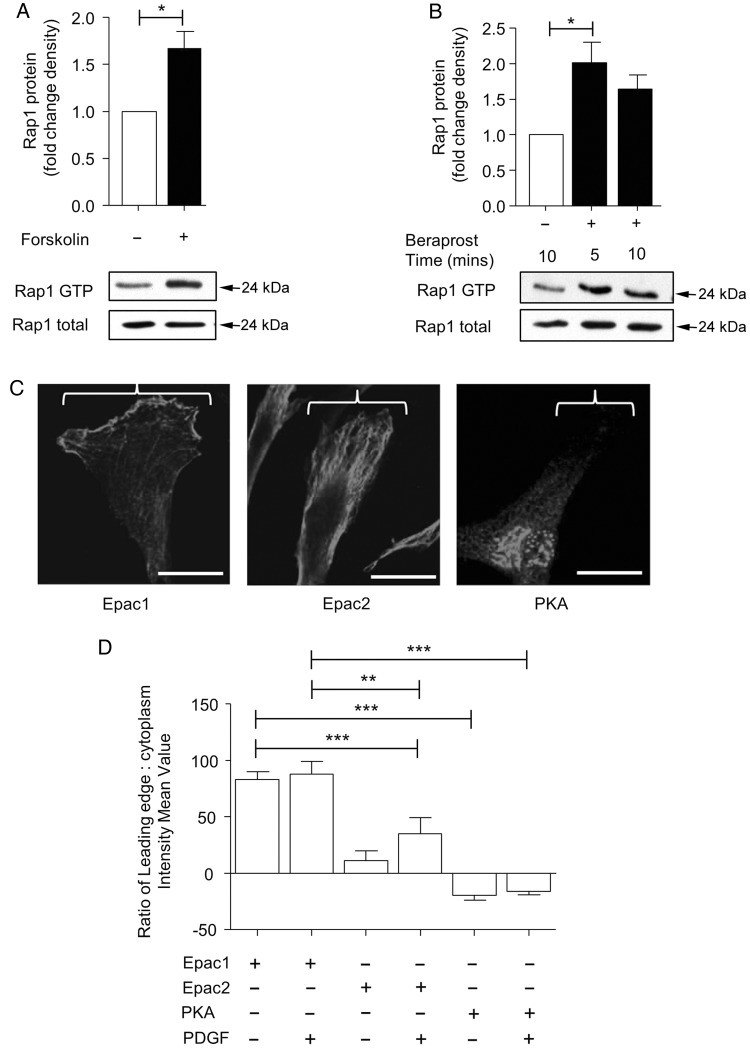
Beraprost activates Rap1 and Epac1 localises to the leading edge. (*A*) Incubation of 10 µM forskolin significantly increased Rap1 association with GTP within 5 min. Data are displayed as Rap1 GTP/total Rap1, mean ± SEM, *n* = 4. (*B*) Rap1 GTP was also significantly increased in following incubation with of 1 nmol/L beraprost after 5 min. *n* = 4. (*C*) Epac1 is predominantly localized to the leading edge in migrating VSMC stimulated for 24 h with PDGF. Epac2 localization occurs throughout the cell and the PKA is localized predominantly at perinuclear sites, *n* = 3. Images show representative cells, scale bar represents 5 µm. (*D*) The location of Epac1, Epac2, and PKA were assessed by intensity measurements. Locations for each protein were confirmed and did not change following PDGF incubation. Data are expressed as the intensity mean value of the leading edge in comparison to the cytoplasm of the VSMC, *n* = 3, *P < 0.05, **P < 0.01, ***P < 0.001.

The above results suggest that beraprost inhibits migration via activation of the Epac/Rap1 pathway. To ensure that beraprost activated Rap1 in human saphenous vein VSMC, we used a pull-down assay specific for GTP-bound Rap1. Forskolin induced an increase in Rap1 activation as determined by elevated GTP binding within 5 min of incubation (*Figure 5A*). Beraprost also resulted in Rap1 activation by ∼2-fold compared with control after 5 min incubation. This remained elevated at 10 min but was not significant (*Figure 5B*).

### 3.5 Epac1 is expressed at the leading edge of VSMC whereas PKA is present at perinuclear locations

To determine which isoforms of Epac are expressed in human saphenous vein VSMC, we performed immunocytochemistry using specific antibodies for Epac1 and Epac2. Immunoblots and real-time PCR determined that Epac1 and Epac2 are expressed in these cells (Supplementary material online, *Figure S3*). VSMC on coverslips were subjected to a scratch and left for 24 h to induce migration. Cells migrating towards the scratch centre with a clearly visible leading edge were analysed. Epac1 was located predominantly at the leading edge (*Figure 5C*). Epac2 had a more even distribution throughout the cell although it was also present at the leading edge. PKA, however, was found almost exclusively in the perinuclear region. Incubation with PDGF did not alter the localization of Epac1, Epac2 or PKA (*Figure 5D*). Beraprost treatment also did not change the distribution of these proteins (Supplementary material online, *Figure S4*).

### 3.6 Beraprost prevents actin cytoskeleton re-arrangement at the leading edge during migration by inhibiting RhoA

**Figure 6:**
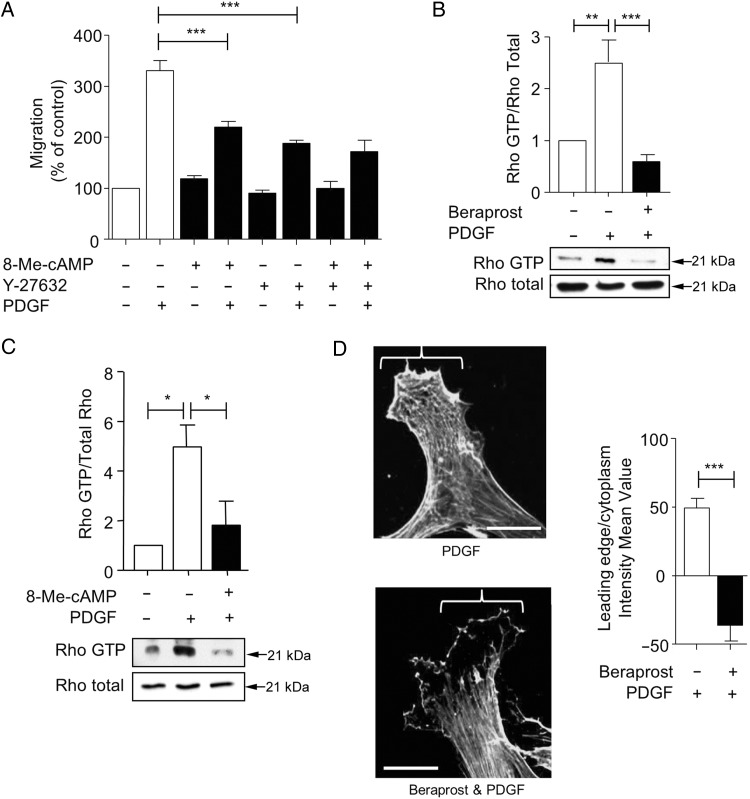
Beraprost inhibits RhoA activation and leads to inhibition of F-actin re-arrangement. (*A*) PDGF-induced migration of VSMC in the chemotaxis chamber was inhibited in the presence of Y-27632 (1 µM) or 8-Me-cAMP (2 µM); however, there was no additional inhibition of migration when VSMC were treated with Y-27632 and 8-Me-cAMP in combination. Migration data are expressed as a % of control, *n* = 3. (*B*) Using a Rhotekin pull-down assay, PDGF incubation for 30 min significantly increased GTP-bound RhoA. Simultaneous incubation with 1 nmol/L beraprost significantly decreased Rho-GTP compared with PDGF alone. Data are expressed as Rho-GTP/Rho total, *n* = 4. (*C*) Incubation with 2 µM 8-Me-cAMP significantly inhibited PDGF-induced Rho-GTP binding in VSMC as assessed by Rhotekin pull-down assay. Data are expressed as Rho-GTP/Rho total, *n* = 3. (*D*) VSMC incubated with PDGF had extensive F-actin expression at the leading edge. Addition of 1 nmol/L beraprost inhibited F-actin formation at the leading edge in comparison to PDGF alone. Representative images are shown. Data are expressed as the intensity mean value of the leading edge in comparison to the cytoplasm, *n* = 3. *P < 0.05, **P < 0.01, ***P < 0.001. Scale bar = 5 µm.

As therapeutic concentrations of beraprost activated the Epac /Rap1 pathway, we examined potential downstream mechanisms which might contribute to the inhibitory effect on migration. Rap1 has previously been shown to inhibit RhoA in several cell types including some smooth muscle cell types.^21^ We firstly examined the role of RhoA in migration of human saphenous vein VSMC. The Rho-kinase inhibitor Y27632 (1 µmol/L) had a similar inhibitory effect on PDGF-induced migration compared with 8-Me-cAMP (*Figure 6A*). This effect obtained at 1 µmol/L Y27632 was submaximal (Supplementary material online, *Figure S5*). This suggests that activation of the RhoA/Rho-kinase pathway is required for PDGF-induced migration. The effects of Y27632 and 8-Me-cAMP combined were not additive. Using Rhotekin pull-down assays specific for GTP-bound RhoA, we observed that PDGF activated RhoA in human VMSC over a 30 min timecourse (*Figure 6B*). Co-incubation of the cells with 1 nmol/L beraprost significantly inhibited RhoA activation. In addition, 8-Me-cAMP inhibited PDGF-induced RhoA activation at the same concentration used to inhibit VSMC migration (*Figure 6C*). As a comparison, the effect of the PKA agonist 6-Phe-cAMP on PDGF-induced RhoA activation was also examined (Supplementary material online, *Figure S6*). Direct PKA stimulation also inhibited RhoA activation, although this effect will not be involved in beraprost-induced inhibition of migration (see *Figure 3*). As RhoA is an important regulator of the actin cytoskeleton, we determined whether beraprost might prevent PDGF-induced effects on F-actin. F-actin filaments observed at the leading edge in PDGF-treated cells, as assessed using immunolocalization, were significantly decreased following co-incubation with 1 nmol/L beraprost (*Figure 6D*).

## 4. Discussion

Preventing migration of VSMC after angioplasty/stenting or vein grafting following bypass surgery is a promising but untested therapeutic target. Drugs which target VSMC migration would decrease the growth of neointima and thereby reduce restenosis. In the current study, human saphenous vein VSMC was examined *in vitro* to determine the potential of cAMP-producing drugs to inhibit VSMC migration. Beraprost, a prostacyclin analogue, was studied as this has already been indicated as potentially useful in some cardiovascular disease states and has therefore direct clinical relevance.^27^ This was assessed at therapeutically relevant concentrations comparable with those achievable in patient plasma.^27^ We reveal that beraprost can inhibit growth-factor-induced VSMC migration at these therapeutic concentrations. The mechanisms involved in this inhibitory effect occur via activation of the cAMP-activated binding protein Epac but do not involve the well characterized and more typical cAMP effector PKA. In our experiments, PKA was not activated by beraprost at clinical concentrations and PKA siRNA did not inhibit the beraprost-induced anti-migratory response. These results demonstrate that cAMP-producing agonists such as beraprost may help slow restenosis following angioplasty by inhibiting VSMC migration through Epac activation.

Until very recently, the functional roles of Epac in VSMC were unknown. Studies using Epac selective activators have now indicated that Epac may be involved in the vasculoprotective effects of cAMP, such as vasorelaxation of VSMC and an inhibition of VSMC proliferation.^21–23^ Activation of Epac via either a direct Epac activator or a cAMP-producing membrane receptor agonist, such as isoproterenol, can produce a vasodilatory effect. This occurs via an increase in Rap1 activity and subsequent inhibition of RhoA resulting in calcium desensitization of myosin II phosphorylation.^21^ In addition, Epac can also activate calcium-sensitive potassium channels in arteries leading to hyperpolarization which decreases calcium entry via voltage-gated calcium channels and also results in vasodilation.^22^ It is not clear whether this effect on potassium channels is dependent on Rap1 signalling. Additionally, Epac can inhibit proliferation in rat and human VSMC.^23,28^ This effect, at least in rat VSMC, is synergistic with PKA as Epac activation alone was not sufficient to inhibit the proliferative effect.^22^ In the present study we did not detect any inhibition of human VSMC proliferation, even at relatively high concentrations of the cAMP-producing agonist beraprost, which can activate both PKA and Epac. However, our current study now indicates that Epac activation (in the absence of PKA activation) is sufficient to inhibit human VSMC migration. A recent contradictory study has indicated that a synthetic Epac activator enhances rat VSMC migration, although therapeutically relevant concentrations of cAMP, such as those generated by cAMP-producing agonists, were not assessed.^29^ This is in contrast to most studies which indicate a protective role for cAMP signalling in the vasculature. We have also examined migration in rat VSMC and although no enhancement was observed with a synthetic Epac agonist, we also could not reproduce our results with regard to the inhibitory effects on human VSMC migration (results not shown). This suggests that there are clear species differences which will be important in determining translational applicability. Regardless, we provide evidence here that Epac is a novel drug target which could potentially be exploited to limit restenosis.

An important finding of this study is that Epac and PKA are differentially regulated in VSMC by varying the concentration of a cAMP-producing agonist. At lower beraprost concentrations (directly comparable to those achieved *in vivo*), only Epac is activated while at higher beraprost concentrations both Epac and PKA are activated. This indicates that lower levels of cAMP production lead to Epac activation alone, although this was not directly measured in our study. Such data correspond with that previously observed in cardiac fibroblasts and provides further evidence for the relevant physiological cAMP affinities for Epac vs. PKA.^19^ Other than these potential differences in cAMP affinity, we have also uncovered further evidence which indicates divergent functions of Epac and PKA. Our study demonstrates that Epac1 is localized predominantly at the leading edge of migrating VSMC cells and is therefore situated at the main site for directional migration. This is in contrast to PKA which has a perinuclear localization relatively distant from both the leading and trailing edge. These distinct localizations may indicate a compartmentalization of signalling with regard to these mediators. Both the potentially lower cAMP affinity together with differences in localization indicate that Epac1 may be more likely (compared with PKA) to be part of the regulation of migration, acting as a brake on leading edge progression.

**Figure 7:**
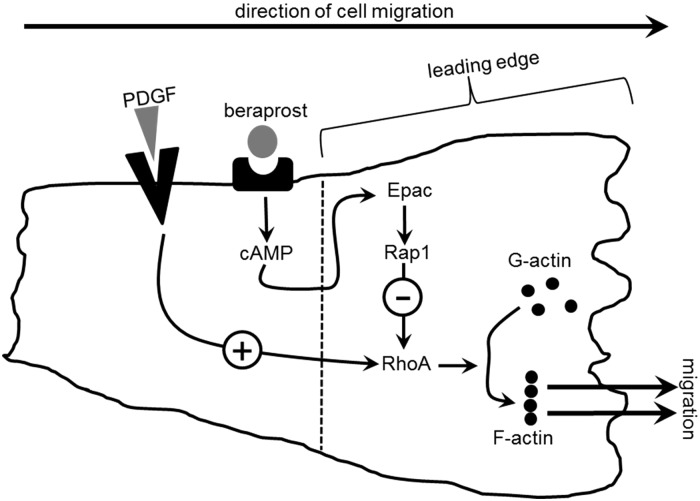
Schematic diagram of proposed mechanism of action of beraprost-induced inhibition of VSMC migration. PDGF produces migration via activation of RhoA resulting in a rearrangement of actin filaments. Beraprost, acting on IP receptors, stimulates cAMP production activating Epac, which in turn leads to GTP-binding of Rap1. GTP-bound Rap1 inhibits RhoA preventing actin reassembly and ultimately decreasing migration.

Although studies have demonstrated the potential importance of Epac in VSMC function, the downstream signalling of Epac is highly relevant to fully understand the mechanisms involved. We reveal that the inhibition of migration induced by Epac is dependent on the subsequent activation of Rap1 which in turn inhibits RhoA activation. The RhoA/Rho-kinase pathway is essential for PDGF-induced migration in human VSMC and is a well-described regulator of the cell cytoskeleton.^4^ We have also demonstrated that Epac activation inhibits migration by preventing rearrangement of the actin cytoskeleton at the leading edge (the site of Epac1 localisation). This likely occurs via Rap1-mediated inhibition of RhoA. Evidence for an important role of Rap1 in VSMC function has been recently demonstrated using Rap1b−/− mice. These mice develop hypertension in part via functional changes to VSMC and this indicates an essential role of Rap1 (and possibly Epac) in maintaining normal vascular physiology.^30^ With regard to the Rap1-induced inhibition of RhoA, other studies have previously demonstrated this link although not in relation to VSMC migration.^21^ We now provide evidence of the importance of this Rap1/RhoA mechanism in a pathologically relevant process and this is summarized in *Figure 7*.

An important part of our study has demonstrated that the clinically approved drug beraprost activates Epac at therapeutically relevant concentrations. Orally-active beraprost is currently used in the treatment of pulmonary hypertension in some countries and may have clinical benefits in peripheral vascular disease.^27^ It must be acknowledged that, in addition to the short half-life, beraprost also has side-effects that could preclude this drug from becoming a routine option following angioplasty.^31^ However, recent developments in technology, including drug-eluting stents and encapsulation of beraprost in slow release nanoparticles, now provide a route of administration which results in more targeted action with lower systemic doses required.^3,32^ Such improvements in drug delivery would decrease side-effects and increase its therapeutic potential. Beraprost is generally cardioprotective via its vasodilatory and anti-platelet effects. It is a prostacyclin analogue which binds to prostanoid receptors leading to activation of adenylate cyclase via the heterotrimeric G-protein, *G*_s_. It has a relatively short half-life *in vivo* and therefore the maximum concentration which can be achieved in plasma is limited to ∼1 nmol/L.^27^ We now demonstrate in human VSMC that this therapeutically relevant concentration of beraprost activates Epac. This suggests that many of the clinically beneficial effects may in fact arise from Epac activation. Our results also indicate that beraprost activation of Epac/Rap1 may be useful in preventing restenosis following angioplasty by blocking VSMC migration. Indeed a recent study has examined the effects of beraprost in an *in vivo* model of in-stent restenosis.^12^ These authors have demonstrated that intravenous beraprost treatment during stent emplacement surgery significantly limited neointimal thickening after 4 weeks although the intracellular mechanisms were not assessed. Our results now suggest that an inhibition of migration may explain the observed beneficial effects of prostacyclin analogues *in vivo*.^12^ Together these data provide evidence of a potential therapeutic benefit of this drug target in restenosis.

In conclusion, we have revealed, for the first time, that therapeutically relevant concentrations of the prostacyclin analogue beraprost can inhibit VSMC migration via activation of the cAMP effector Epac. The mechanism of this Epac-mediated inhibition is via activation of Rap1 and subsequent inhibition of RhoA preventing actin cytoskeletal changes. This indicates that cAMP-producing agonists such as beraprost may be beneficial following angioplasty to prevent neointimal hyperplasia.

## Supplementary material

Supplementary material is available at *Cardiovascular Research* online.

**Conflict of interest:** none declared.

## Funding

This work was supported by the British Heart foundation (grant FS/11/23/28730). J.S.M. was funded by a British Heart Foundation PhD studentship. Funding to pay the Open Access publication charges for this article was provided by the Charities Open Access Fund (UK).
